# Study on Multimodal Sensor Fusion for Heart Rate Estimation Using BCG and PPG Signals

**DOI:** 10.3390/s26020548

**Published:** 2026-01-14

**Authors:** Jisheng Xing, Xin Fang, Jing Bai, Luyao Cui, Feng Zhang, Yu Xu

**Affiliations:** College of Electrical and Information Engineering, Beihua University, Jilin 132021, China; jisheng_xing@beihua.edu.cn (J.X.); fxxxxx0712@163.com (X.F.); jlbyj@beihua.edu.cn (J.B.); cuiluyao16@gmail.com (L.C.); 18037388227@163.com (F.Z.)

**Keywords:** ballistocardiography, photoplethysmography, multimodal fusion, temporal convolutional network, heart rate monitoring, contactless monitoring

## Abstract

Continuous heart rate monitoring is crucial for early cardiovascular disease detection. To overcome the discomfort and limitations of ECG in home settings, we propose a multimodal temporal fusion network (MM-TFNet) that integrates ballistocardiography (BCG) and photoplethysmography (PPG) signals. The network extracts temporal features from BCG and PPG signals through temporal convolutional networks (TCNs) and bidirectional long short-term memory networks (BiLSTMs), respectively, achieving cross-modal dynamic fusion at the feature level. First, bimodal features are projected into a unified dimensional space through fully connected layers. Subsequently, a cross-modal attention weight matrix is constructed for adaptive learning of the complementary correlation between BCG mechanical vibration and PPG volumetric flow features. Combined with dynamic focusing on key heartbeat waveforms through multi-head self-attention (MHSA), the model’s robustness under dynamic activity states is significantly enhanced. Experimental validation using a publicly available BCG-PPG-ECG simultaneous acquisition dataset comprising 40 subjects demonstrates that the model achieves excellent performance with a mean absolute error (MAE) of 0.88 BPM in heart rate prediction tasks, outperforming current mainstream deep learning methods. This study provides theoretical foundations and engineering guidance for developing contactless, low-power, edge-deployable home health monitoring systems, demonstrating the broad application potential of multimodal fusion methods in complex physiological signal analysis.

## 1. Introduction

Cardiovascular diseases (CVDs) represent the leading cause of mortality worldwide, making their early detection and prevention critical priorities in public health [1,2]. According to data published by the Journal of the American College of Cardiology, CVDs accounted for 19.8 million deaths globally in 2022, with particularly rapid increases in disease burden observed in developing countries [3]. In China, where the population is aging rapidly, over 330 million people are affected by CVDs, and the prevalence of hypertension, coronary artery disease, and arrhythmias continues to rise annually [2]. This trend underscores the urgent need for long-term, continuous, and accurate heart rate monitoring to support early disease detection and personalized health management.

Although electrocardiography (ECG) is considered the gold standard for heart rate monitoring, its reliance on adhesive electrodes often causes skin irritation and restricts daily activities, rendering it unsuitable for unobtrusive, long-term monitoring in home environments [4]. To meet this need, wearable photoplethysmography (PPG) has emerged as the standard technology integrated into smartwatches, providing a vital means for routine heart rate monitoring. However, PPG technology possesses inherent limitations when applied to precise and continuous monitoring required for the early detection and management of cardiovascular diseases. The signal is highly susceptible to motion artifacts, which degrades measurement reliability during physical activity. More critically, as an indirect measure of cardiac activity via peripheral pulse waves, PPG exhibits a time delay relative to the heart’s electrical activity. This delay is dictated by pulse transit time (PTT), which is dynamically modulated by vascular tone and blood pressure. Such physiological variability can induce significant measurement discrepancies, particularly during rapid heart rate fluctuations or arrhythmic events [5]. Furthermore, measurement accuracy is significantly influenced by physiological variables such as skin tone, age, and vascular health, which restricts the generalizability and reliability of technology across diverse populations [6].

These limitations have accelerated the development of contactless sensing technologies, particularly ballistocardiography (BCG), which capture cardiac mechanical activity through subtle body vibrations using high-sensitivity sensors such as piezoelectric films, fiber-optic arrays [7], or accelerometers [8]. BCG can be seamlessly integrated into everyday objects such as mattresses or chairs to enable 24 h continuous monitoring without the discomfort associated with traditional ECG electrodes. Furthermore, BCG signals provide multidimensional insights into cardiac function, offering simultaneous information regarding heart rate, myocardial contractility, and cardiac output [9,10]. Despite its promise, BCG signal acquisition presents significant challenges. The signals are extremely weak and highly susceptible to noise from respiration, muscle movement, and external vibrations. Signal quality is strongly influenced by sensor placement and user posture, resulting in considerable variation in waveform morphology. Unlike ECG, which exhibits relatively standardized waveforms, BCG demonstrates high inter-individual variability and lacks universal morphological standards. In shared or noisy environments, reliable signal separation and accurate recognition remain critical technical challenges.

To address these limitations, this study introduces a novel multimodal temporal fusion network (MM-TFNet) that exploits the time-series characteristics of physiological signals. MM-TFNet represents the first architecture to integrate temporal convolutional networks (TCNs), bidirectional long short-term memory (BiLSTM), and multi-head self-attention mechanisms to enable collaborative feature learning and dynamic weighting of BCG and PPG signals. The model employs multi-scale dilated causal convolutions with hierarchical residual connections to capture both local and long-range temporal dependencies. Additionally, the combination of BiLSTM and attention mechanisms enhances sequential modeling and enables dynamic focus on critical signal components. An adaptive cross-modal fusion layer learns attention weights that optimize the complementary integration of BCG and PPG features. The overall architecture adopts a pyramid-style feature extraction strategy, enhanced with spatial dropout and multi-scale receptive field fusion, achieving robust performance under complex physiological conditions.

The structure of this paper is as follows. Section 2 surveys existing methodologies. Section 3 describes the experimental methodology, while Section 4 details the data collection and processing procedures. Section 5 introduces the evaluation method and experimental results in detail and further compares them with methods proposed in other literature. Discussion and conclusion are given in Section 6 and Section 7, respectively.

## 2. Related Work

Significant progress has been made in single-modal sensing techniques for BCG signal analysis. Early research on heart rate estimation using BCG primarily relied on conventional signal processing methods, extracting waveform features from the time or frequency domains to derive physiologically meaningful insights. Brink et al. [11] employed a peak detection approach, using a fixed threshold to identify J-wave peaks as indicators of heartbeat locations. As digital signal processing advanced, various algorithms were introduced. Heise et al. [12,13] proposed a sliding window method that estimates heart rate by calculating the difference between the most negative and most positive samples within each window. Although straightforward and computationally efficient, this approach performs poorly under low signal-to-noise conditions. Xie et al. [14] applied template matching, aligning real-time BCG signal s with a reference heartbeat template to detect cardiac cycles. Additionally, methods such as autocorrelation [15], cepstral analysis [16], and short-time Fourier transform (STFT) [17] have been widely employed in BCG processing. However, these techniques continue to face considerable challenges when handling signals corrupted by noise or respiratory modulation.

Machine learning has introduced new perspectives for BCG signal analysis. Rosales et al. [18] pioneered the use of K-means clustering to extract features from BCG signals, categorizing signal segments into heartbeat and non-heartbeat classes for accurate beat detection. Similarly, Paalasmaa et al. [19] proposed a clustering-based template learning method that improved heart rate estimation by adaptively refining heartbeat templates. While these approaches perform well with clean, standardized BCG waveforms, they often struggle to handle signals distorted by changes in body position or motion artifacts. The rapid development of deep learning in recent years has brought transformative advancements to BCG signal processing. Cathelain et al. [20] were the first to apply a U-Net architecture to BCG heartbeat detection, enabling precise identification of IJK waveforms and providing a novel direction for contactless heart rate monitoring. Jiao et al. [21] proposed a BiLSTM based regression network that estimates heart rate without relying on exact beat annotations, instead learning temporal patterns directly from raw BCG data, thereby effectively reducing the model’s dependency on signal quality. Building on this work, Mai et al. [22] integrated U-Net with bidirectional LSTM in an end-to-end detection framework, which demonstrated excellent robustness under low signal-to-noise conditions. Convolutional neural networks (CNNs) have also been widely adopted in BCG analysis. Pröll et al. [23] compared various deep learning architectures and found that hybrid CNN-RNN models were more effective at capturing the spatiotemporal features of BCG signals, outperforming conventional methods by a substantial margin. Su et al. [24] further introduced a multi-scale attention-based CNN, where attention mechanisms were employed to dynamically learn feature importance, thereby enhancing the accuracy of relative blood pressure estimation. Most recently, Zhang et al. [25] proposed the first Conv-Transformer model for heart rate estimation from BCG signals. Their approach combines local feature extraction via CNNs with global dependency modeling through Transformers, enabling end-to-end heart rate prediction without requiring post-processing. Heart rate estimation based on single-modal BCG signals has progressed significantly, evolving from traditional signal processing techniques to advanced deep learning methods. These modern approaches can automatically extract relevant features and have substantially improved the accuracy and robustness of heart rate detection, particularly in addressing challenges such as noise suppression, heartbeat localization, and real-time estimation [4,26].

However, relying solely on BCG signals remains insufficient in complex real-world environments. Issues such as motion-induced signal distortion, individual physiological variability, and waveform alterations under pathological conditions continue to limit performance.

These challenges have driven growing interest in multimodal fusion strategies. Recent research has explored the fusion of data from multiple sensors using diverse methodologies. For example, John et al. [27] employed discrete wavelet transform to decompose ECG and PPG signals and then fused the resulting wavelet coefficients through weighted averaging. Their method outperformed single-channel approaches across multiple experimental settings. This cross-modal strategy is equally applicable to the integration of BCG and PPG signals, which are physiologically complementary—similar to the relationship between ECG and PPG. Specifically, BCG reflects mechanical cardiac activity by capturing subtle body vibrations caused by heart contractions, while PPG records optical changes associated with blood volume dynamics in peripheral vessels. These two modalities provide different yet temporally correlated perspectives on cardiac function, and their combined use can enhance both the robustness and accuracy of heart rate monitoring. Feature-level fusion of BCG and PPG signals can improve noise resistance and yield a more comprehensive understanding of the cardiac cycle. For instance, Warnecke et al. [28] employed a CNN-based approach to integrate ECG, BCG, PPG, and image-based PPG (iPPG) signals for robust heartbeat detection in automotive environments. Their results demonstrated that multisensor fusion significantly improves the reliability of heart rate monitoring. These findings confirm the practical feasibility of multimodal fusion, especially in applications requiring comfort and non-invasiveness, such as wearable devices and smart bedding systems.

To provide a clear comparative overview of the evolution and performance of BCG-based heart rate estimation methods, a summary of representative approaches is presented in Table 1.

## 3. Methods

The design of the MM-TFNet is grounded in the complementary physiological nature of BCG and PPG signals within the cardiac cycle. The BCG signal originates from the mechanical recoil forces generated by ventricular contraction and blood ejection, capturing central events such as the opening of the aortic valve. In contrast, the PPG signal arises from subsequent volumetric changes in peripheral microvascular bed due to the arriving pressure pulse wave. Although BCG and PPG are temporally correlated, a physiological delay exists between them known as PTT. This complementary relationship means that BCG provides a more direct measure of the heartbeat’s mechanical initiation, while PPG offers a clearer waveform morphology of the peripheral pulse. By jointly modeling these aligned yet different signals, the fusion model can construct a more robust representation of each cardiac cycle. Specifically, BCG can anchor the timing of heartbeats, while PPG can refine the detection, thereby enhancing the model’s overall resistance to interference and mitigating motion artefacts that could potentially affect any single modality individually.

Aiming to harness this complementarity, we proposed the MM-TFNet architecture. This model integrates TCN, BiLSTM and MHSA to achieve dynamic, feature-level fusion of BCG and PPG signals, thereby enabling accurate heart rate estimation.

### 3.1. TCN

TCN is a convolutional architecture specifically designed for modeling sequential data, capable of capturing long-range temporal dependencies while maintaining high computational efficiency [29]. TCNs have been successfully applied across various domains, including traffic forecasting, speech recognition, machine translation, and wind power prediction. However, their application to BCG signal-based regression tasks remains largely unexplored.

In this study, we employ a TCN-based framework to extract temporal features from BCG and PPG signals and leverage feature fusion to enable accurate heart rate prediction. The key components of the TCN architecture include causal convolution, dilated convolution, and residual connections.

Causal convolution ensures that each output at time step t depends only on current and past inputs, preserving temporal causality and preventing information leakage from future time steps. It is formally defined as Equation (1):

(1) $$F\left(t\right)=\sum_{i=0}^{k-1}f\left(i\right)\cdot x_{t-i},$$

where F(t) denotes the output at time t, k is the kernel size, f(i) represents the weight of the i-th element in the convolution filter, and x_t−i_ is the input value at time t − i, corresponding to current and past inputs.

**Figure 1:**
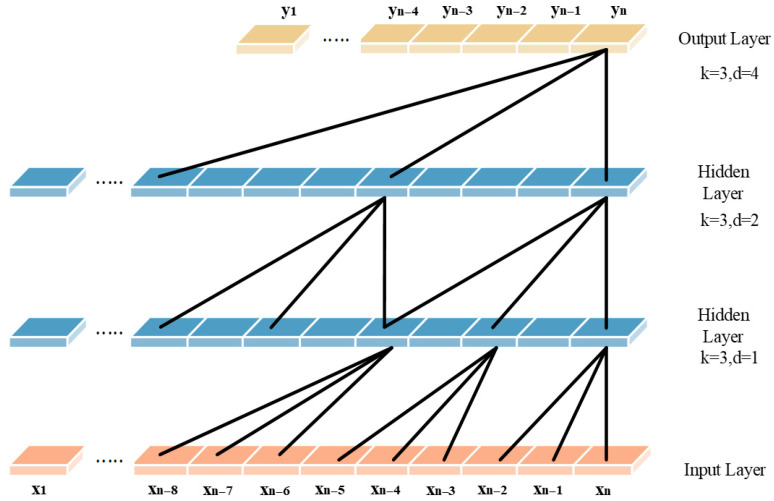
Dilated causal convolution of the TCN.

Dilated convolution expands the receptive field exponentially, enabling the model to learn temporal patterns across multiple time scales without significantly increasing parameters. It is defined as Equation (2):

(2) $$F\left(s\right)=\left(x{*}_{d}f\right)\left(s\right)=\sum_{i=0}^{k-1}f\left(i\right)\cdot x_{s-di},$$

where F(s) is the output at position s, d is the dilation factor controlling the spacing between inputs, and k is the kernel size. Common dilation rates follow powers of two, such as 1, 2, 4, 8. Its structure is shown in Figure 1.

**Figure 2:**
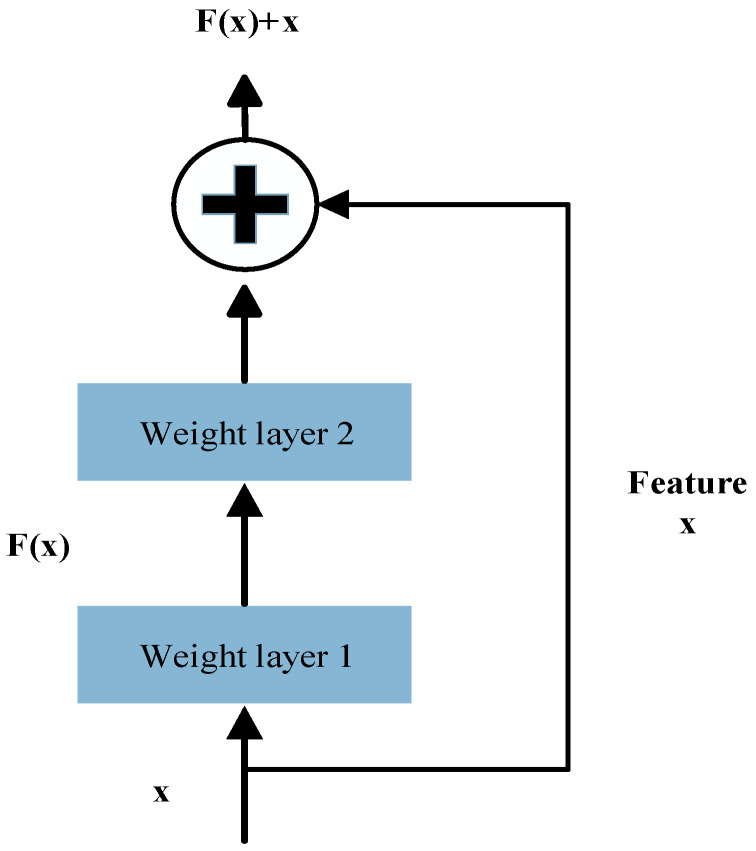
The TCN residual block structure.

TCN incorporates residual connection modules like those in ResNet, which promote stable gradient propagation and enable the construction of deeper network architectures, thereby effectively mitigating the vanishing gradient problem. As illustrated in Figure 2, each residual block contains a branch that passes through a series of transformations denoted as F, the output of which is added to the block’s original input x.

The transformation can be expressed as Equation (3):

(3) $$o=\mathrm{ReLU}\left(x+F\left(x\right)\right),$$

Here, F represents the residual mapping function, which consists of two dilated causal convolution layers followed by nonlinear activation functions and employs a 1 × 1 convolution to align input and output dimensions.

### 3.2. BiLSTM

**Figure 3:**
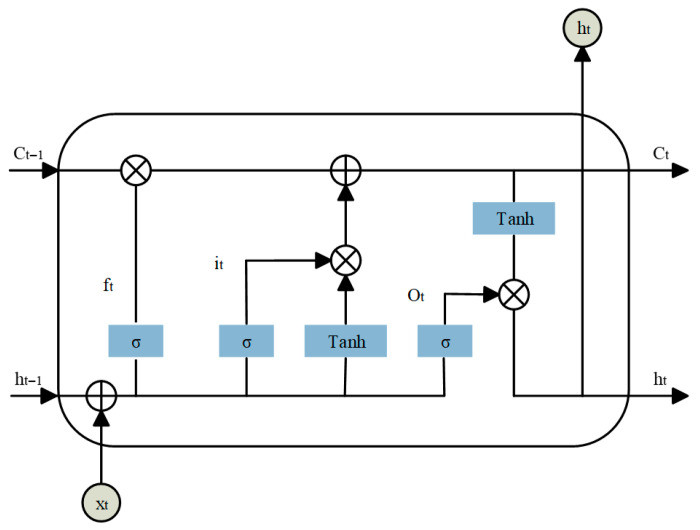
LSTM network unit.

Long Short-Term Memory (LSTM) is an advanced form of recurrent neural network (RNN) specifically designed to handle sequential data. It effectively addresses the vanishing and exploding gradient problems that often limit the performance of traditional RNNs. From the structural diagram in Figure 3, we can see that an LSTM unit comprises three key gates: the forget gate, the input gate, and the output gate. The forget gate determines the proportion of historical information to retain, filtering out redundant or outdated features. The input gate controls how new information is integrated into the memory cell, embedding important transformations from the input signal. The output gate extracts relevant information from the updated memory and generates a hidden state for downstream predictions.

The computations within an LSTM unit can be described as Equations (4)–(9):

(4) $$f_{t}=\sigma \left(W_{f}x_{t}+U_{f}h_{t-1}+b_{f}\right),$$

(5) $$i_{t}=\sigma \left(W_{i}x_{t}+U_{i}h_{t-1}+b_{i}\right),$$

(6) $$o_{t}=\sigma \left(W_{o}x_{t}+U_{o}h_{t-1}+b_{o}\right),$$

(7) $${\tilde{C}}_{t}=\tanh(W_{c}x_{t}+U_{c}h_{t-1}+b_{c}),$$

(8) $$C_{t}=f_{t}\cdot C_{t-1}+i_{t}\cdot {\tilde{C}}_{t},$$

(9) $$h_{t}=o_{t}\cdot \tanh(C_{t}),$$

In these equations, x_t_ is the input vector, ft, it, and o_t_ denote the forget, input, and output gates, respectively. c_t_ represents the cell state, and σ is the activation function for the gates. W, U, and b are learnable weight matrices and bias vectors. h_t_ denotes the hidden state vector, also referred to as the output of the LSTM unit.

**Figure 4:**
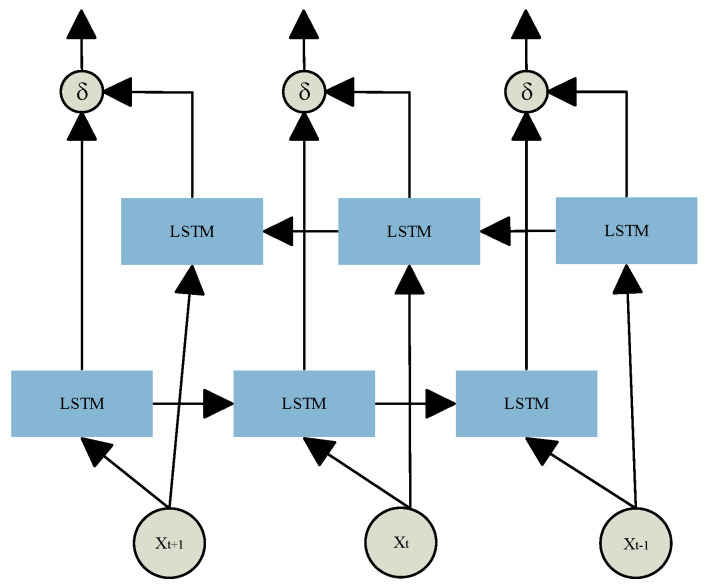
BiLSTM structure.

These gate mechanisms enable LSTM networks to effectively capture long-term dependencies in sequential data while dynamically updating memory to adapt to temporal changes. To enhance temporal modeling, Bidirectional LSTM (BiLSTM) extends the standard LSTM by introducing two independent LSTM layers that process the input sequence in forward and reverse directions. As shown in Figure 4, BiLSTM captures both past and future context within the sequence, enabling a more comprehensive analysis of the dynamic patterns in physiological signals. This dual-path structure overcomes the unidirectional limitations of traditional LSTM.

The BiLSTM operations can be formally expressed as Equation (10):

(10) $$h_{i}=\delta \left({\vec{h}}_{i},{\overset{\leftarrow }{h}}_{i}\right),$$

where ${\vec{h}}_{i}$ and ${\overset{\leftarrow }{h}}_{i}$ represent the sequences of hidden states generated by the LSTM layers in the forward and backward temporal directions, respectively. These are then passed into two separate LSTM layers, and their outputs are combined to produce the final BiLSTM representation h_i_ out.

### 3.3. Multi-Headed Self-Attention

Multi-Head Self-Attention (MHSA) is a key component of the Transformer architecture, designed to capture complex dependencies within sequences. By enabling parallel multi-perspective feature extraction, MHSA significantly enhances the model’s capacity to understand contextual relationships. The core idea is to project the input sequence into multiple distinct subspaces, within which attention mechanisms independently learn the relationships between sequence elements. These subspace-specific features are then aggregated to form a comprehensive global representation. The computation begins by linearly transforming the input sequence X into three matrices: queries Q = XWQ, keys K = XWK, and values V = XWV, where WQ, WK and WV are trainable weight matrices. The outputs are then split into h attention heads, each operating in a separate subspace to compute self-attention independently. The formula is shown in Equation (11).

(11) $${\mathrm{head}}_{i}=\mathrm{Softmax}\left(\frac{Q_{i}K_{i}^{⊤}}{\sqrt{d_{k}}}\right)V_{i},$$

Here, d_k_ denotes the dimensionality of each attention head, and is used to scale the dot-product attention scores to prevent gradient instability. The softmax function normalizes the attention weights to a probability distribution within the range [0, 1]. The outputs from all attention heads are concatenated and passed through a linear transformation to produce the final output. The formula is shown in Equation (12).

(12) $$\mathrm{MHSA}\left(Q,K,V\right)=\mathrm{Concat}\left({\mathrm{head}}_{1},\ldots ,{\mathrm{head}}_{h}\right)W^{O},$$

where W^O^ is the output projection matrix, which maps the concatenated high-dimensional features back to the original model dimension.

### 3.4. Proposed Model Architecture

**Figure 5:**
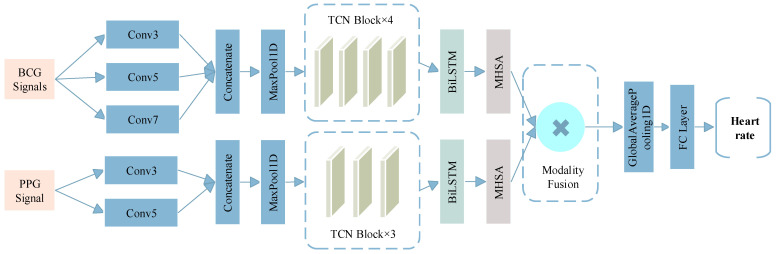
The structure of MM-TFNet.

Building upon the strengths of the previously introduced modules in handling long-range temporal modeling, we propose a novel end-to-end hybrid neural architecture named Multi-Modal Temporal Fusion Network (MM-TFNet). As illustrated in Figure 5, the model integrates a Temporal Convolutional Network (TCN), a Bidirectional Long Short-Term Memory (BiLSTM) network, and a Multi-Head Self-Attention (MHSA) mechanism, employing dynamic feature-level fusion to facilitate hierarchical temporal feature interaction. The model is designed to address three major challenges: (1) enhancing the capture of periodic patterns in physiological signals via multi-scale temporal modeling; (2) developing a dynamic feature selection mechanism to suppress motion artifacts and noise; and (3) achieving complementary cross-modal fusion to improve the robustness of heart rate estimation under complex scenarios.

The network adopts a dual-branch parallel architecture to independently process BCG and PPG signals. In the BCG branch, the input layer receives four-channel BCG signals with 400 sampling points. To cope with signal nonstationarity, a set of multi-scale convolution kernels (3 × 1, 5 × 1, 7 × 1) is employed to capture local waveform variations across different temporal ranges to adapt to the individual differences in the duration of cardiac mechanical events. The resulting feature maps are down sampled via max pooling and fed into a four-stage TCN module with dilation rates of 1, 2, 4, and 8. Each TCN block employs a pre-activation structure combined with spatial dropout to enhance representational capacity while mitigating temporal pseudo-correlations through random channel masking. Similarly, the PPG branch receives single-channel input of 400 sampling points. Given that PPG signals are characterized by simpler unimodal pulses, a lightweight architecture is adopted. A two-layer multi-scale convolution module (3 × 1, 5 × 1) extracts fundamental pulse waveform features, followed by max pooling and a three-stage TCN module with dilation rates of 1, 2, and 4.

The extracted features from both branches are fed into a BiLSTM layer to capture cross-timestep contextual dependencies, followed by a four-head self-attention mechanism to enhance the salience of key temporal features. To prevent mismatches between feature dimensions and attention head counts, the model dynamically adjusts the number of heads, ensuring even division of the feature space and improved computational efficiency. In the fusion stage, we introduce a dual-path projection attention mechanism. Since features extracted from the BCG and PPG branches typically differ in semantic and dimensional properties, direct concatenation may lead to conflicts or redundancy. Therefore, both modalities are first projected into a unified feature space via fully connected layers, using the following transformation, The formula is shown in Equations (13) and (14).

(13) $$P_{\mathrm{BCG}}=W_{\mathrm{BCG}}^{\mathrm{proj}}\cdot F_{\mathrm{BCG}}+b_{\mathrm{BCG}}^{\mathrm{proj}},$$

(14) $$P_{\mathrm{PPG}}=W_{\mathrm{PPG}}^{\mathrm{proj}}\cdot F_{\mathrm{PPG}}+b_{\mathrm{PPG}}^{\mathrm{proj}},$$

where W^proj^ and b^proj^ are learnable parameters. To enable dynamic feature selection, a cross-modal attention mechanism computes the importance weights of BCG and PPG features at each timestep. The formula is shown in Equations (15) and (16).

(15) $$S=\mathrm{Softmax}\left(\frac{P_{\mathrm{BCG}}\cdot P_{\mathrm{PPG}}^{⊤}}{\sqrt{D_{\mathrm{fusion}}}}\right),$$

(16) $$F_{\mathrm{fused}}=S\cdot P_{\mathrm{PPG}}+\left(1-S\right)\cdot P_{\mathrm{BCG}},$$

where S∈ℝ^T×T^ denotes the similarity matrix. The fused features are temporally compressed via a global average pooling layer and passed through a fully connected network (128→64→1) for heart rate regression, followed by a linear scaling operation to map predictions to the actual BPM range.

The model is trained for 100 epochs with early stopping enabled (patience = 15) to prevent overfitting. Training is performed using the Nadam optimizer with an initial learning rate of 1 × 10^−3^. A dynamic learning rate scheduler halves the learning rate if the training loss does not improve for five consecutive epochs. The batch size was set to 32, providing a reliable gradient estimate while maintaining computational efficiency on the GPU. Furthermore, the Huber loss function (δ = 1.0) was specifically chosen for its robustness; it acts like MSE for small errors but like MAE for large ones, effectively mitigating the impact of sudden motion-induced outliers common in BCG and PPG signals.

## 4. Data Sources and Preprocessing

### 4.1. Dataset Description

**Figure 6:**
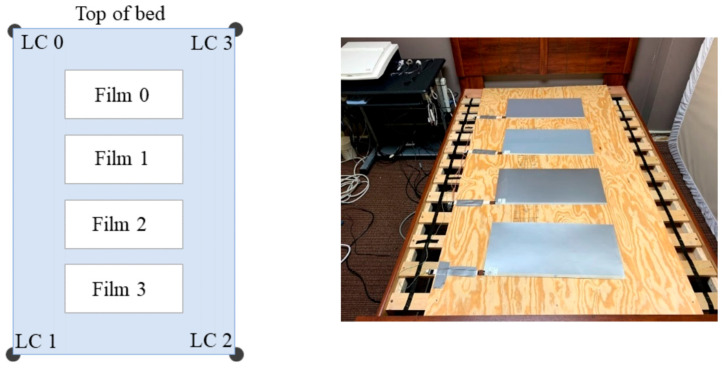
Dataset Sensor Implementation [30].

The dataset used in this study is the publicly available cardiac-driven signal database developed by Carlson et al. [30] at Kansas State University in 2020. This database comprises synchronously recorded BCG, ECG, PPG, and continuous blood pressure waveforms from 40 participants aged 18–65 years. Demographic and anthropometric information for all subjects is summarized in Table 2, including four individuals diagnosed with cardiovascular conditions such as hypertension, atrial fibrillation, or coronary artery disease signals were acquired using a four-channel electromechanical film (EMFi) sensor and a four-channel load cell (LC) sensor positioned beneath a mattress to enable unobtrusive, real-time monitoring. The configuration of the eight sensor channels is illustrated in Figure 6. Simultaneously, ECG and PPG signals were acquired using a GE Datex CardioCap 5 monitor. The BCG signal is processed by a 0.3–24 Hz bandpass filter to suppress environmental noise and respiratory artifacts: the ECG signal is filtered by a 0.5–40 Hz bandpass filter based on prior knowledge. For digitization, the system employed a National Instruments (NI) 9184 Ethernet chassis with NI 9220 modules, which provided synchronous sampling at a sampling frequency of 1 kHz. This setup allows for precise time-alignment with the synchronized ECG and PPG signals recorded by the GE Datex CardioCap 5 monitor (delayed by no more than 15 ms), providing a reliable and certified-standardized reference for deep learning model training. Due to incomplete LC data in one channel for certain subjects, only the four-channel EMFi sensor data were utilized in this study.

### 4.2. Dataset Preprocessing

The raw multi-channel BCG, ECG, and PPG signals were synchronously recorded at a sampling rate of 1 kHz to preserve high-frequency features. To balance computational efficiency with reliable input provision for deep learning models, all signals were subsequently downsampled to 100 Hz. The data were then segmented using a sliding window approach with a 4 s window and 1 s stride. It aims to capture enough 3 to 8 heartbeats to ensure stable heart rate estimation even at low resting heart rates. For each segment, the heart rate was computed and used as the regression target. Specifically, R-peaks were detected from the synchronized ECG signal, and the average RR interval within each window was calculated to derive the corresponding heart rate, which served as the ground truth for supervised training. According to prior statistical analysis and the characteristics of the collected dataset [31], heart rates during rest typically ranged from 35 to 120 beats per minute (bpm). Segments with heart rates outside this physiological range were considered outliers and excluded from analysis. Missing values were subsequently imputed using linear interpolation to minimize computational overhead. Given the considerable inter-subject variability in age, height, weight, and other physiological parameters, significant differences in BCG signal amplitude were observed across participants. Therefore, in this paper, we use z-score to alleviate this difference and ensure that the model learns morphological features rather than absolute signal strength. The normalization was performed using the following Equation (17):

(17) $$z=\frac{\left(x-\mu \right)}{\sigma },$$

where μ denotes the mean value, σ denotes the standard deviation.

## 5. Results

Our study employs a Multi-Modal Temporal Fusion Network (MM-TFNet) to jointly analyze synchronously collected BCG and PPG signals, with systematic validation of the model’s heart rate estimation performance. During training, we prioritized Mean Absolute Error (MAE) as the primary evaluation metric, which provides an intuitive and robust accuracy measure by averaging absolute deviations between predicted and reference heart rates. MAE demonstrates reduced sensitivity to outliers and aligns directly with clinically acceptable error thresholds, establishing a reliable metric for evaluating multimodal signal fusion and algorithmic performance. Unlike existing approaches, MM-TFNet exploits the inherent characteristics of each modality, enhancing periodic pattern capture in physiological signals through multi-scale temporal modeling while improving robustness in complex environments via cross-modal feature integration. The proposed MM-TFNet achieved an MAE of 0.88 BPM, demonstrating highly accurate heart rate predictions.

To check the internal structure of the model, we performed progressive ablation experiments (see Table 3 for details) to evaluate the respective contributions of the core components TCN, BiLSTM and MHSA, and to assess the effectiveness of the fusion strategy. Beginning with a baseline model comprising parallel multi-scale convolution blocks and a dynamic feature fusion module, we incrementally incorporated key modules under identical hyperparameter settings. The TCN module introduction reduced MAE from 3.47 BPM to 3.27 BPM by expanding the receptive field and enhancing long-term temporal feature extraction. Subsequently, adding a BiLSTM layer further decreased MAE to 1.37 BPM, effectively capturing complex temporal dependencies and phase relationships between BCG and PPG signals. Incorporating MHSA reduced MAE to 0.88 BPM by dynamically focusing on critical heartbeat-related features. It is noteworthy that when PPG data is removed and the model relies only on BCG data, or when BCG data is removed and the model relies only on PPG data, performance deteriorates significantly (MAE increases to 1.51 BPM and 3.11 BPM, respectively). This outcome confirms the complementary value of optical and mechanical information. Furthermore, comparative analysis between simple feature concatenation and our dynamic fusion method validated the positive impact of the Modality Fusion (MF) module on model accuracy.

**Figure 7:**
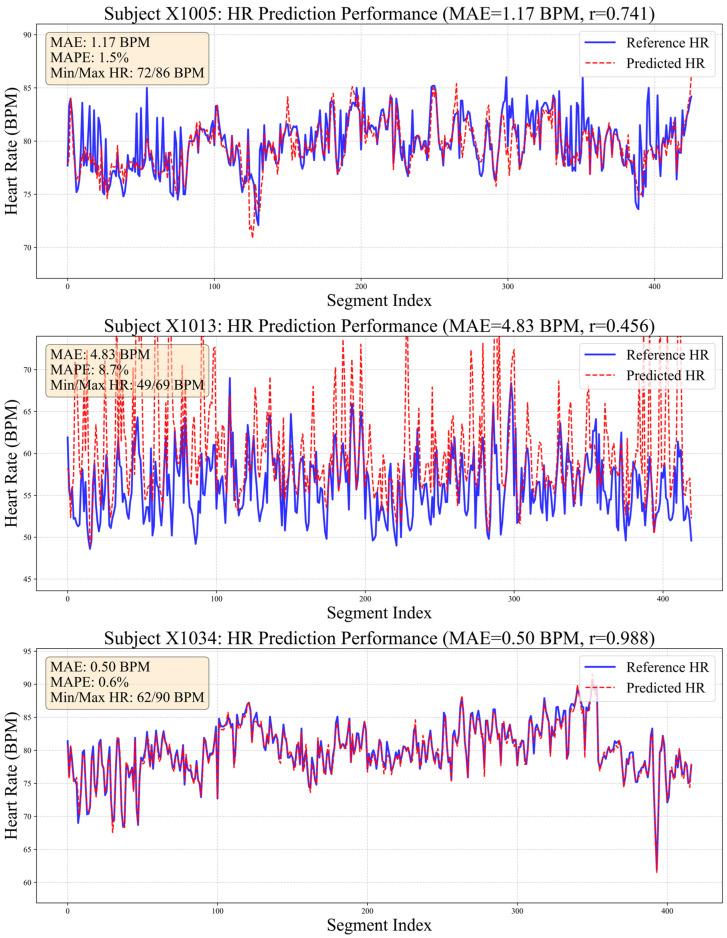
Comparison chart of true and estimated heart rate values.

Beyond primary evaluations, we conducted comparative experiments with alternative heart rate estimation methods to assess our proposed model’s effectiveness. Given that numerous studies have consistently demonstrated deep learning’s superior performance over traditional approaches [32], conventional methods were excluded from this comparison. As summarized in Table 4, our method achieved outstanding results across all evaluated metrics. Figure 7 visualizes HR distribution during resting states for three representative subjects using BCG signals processed by our algorithm.

Notably, 80% of samples achieved MAE below 1 BPM, demonstrating the efficacy of the proposed multi-scale temporal modeling and dynamic feature fusion strategies in enhancing waveform recognition precision. In challenging cases, such as subject X1013 (MAE = 4.83 BPM), ground-truth HR exhibited substantial fluctuation (±12 BPM). This difference highlights the challenge of tracking rapid physiological changes using only mechanical and optical signals and indicates that the model needs stronger prior information during training. For subject X1005, despite slight systematic underestimation in predictions, strong linear correlation with reference values was maintained, indicating reliable capture of relative HR trends.

**Figure 8:**
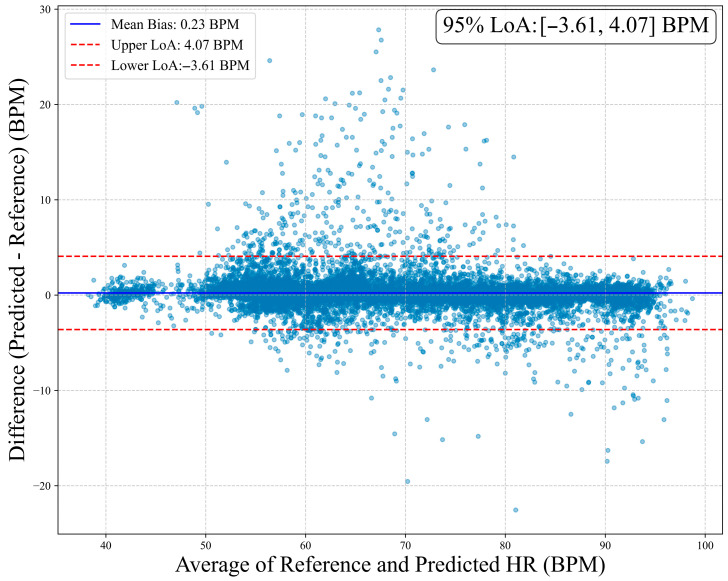
Bland–Altman plot of heart rate estimation. The horizontal axis represents the average of the true values and the predicted values, while the vertical axis shows the difference between the predicted values and the reference values. The plot marks the mean bias (0.23 BPM) and its 95% limits of agreement (upper limit 4.07 BPM, lower limit −3.61 BPM).

**Figure 9:**
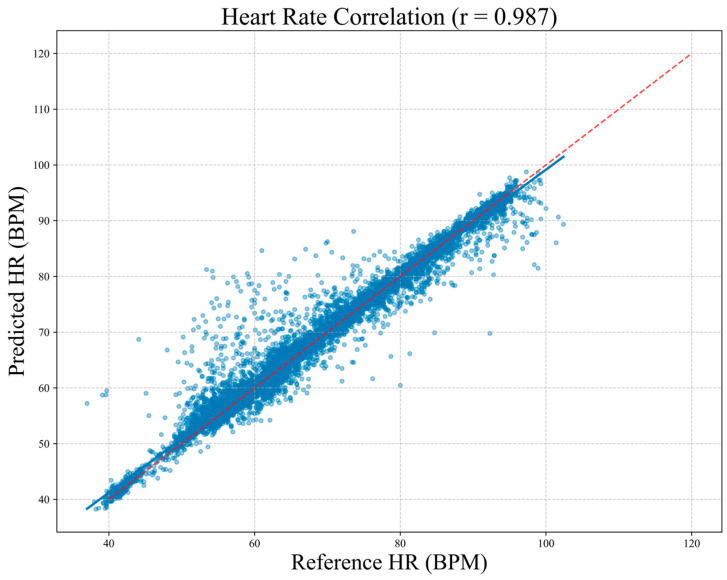
Correlation scatter plot between predicted heart rate and reference heart rate. Each blue point in the plot represents a single measurement, and the diagonal red line denotes the ideal line of agreement (y = x). The Pearson correlation coefficient is r = 0.987, indicating a high level of consistency between the two methods for heart rate measurement.

To assess clinical agreement between MM-TFNet heart rate predictions and ECG-derived reference values, we performed Bland–Altman analysis. As demonstrated in Figure 8, the mean bias was 0.23 BPM, indicating negligible systematic deviation. The 95% limits of agreement ranged from 3.61 BPM to 4.07 BPM, suggesting that most differences fell within clinically acceptable ranges. Figure 9 corroborates these findings, with predicted and reference values demonstrating strong alignment along the identity line (y = x). The high Pearson correlation coefficient (PCC = 0.987) confirms model accuracy and consistency across the complete heart rate spectrum, underscoring strong potential for clinical application.

In order to evaluate the feasibility of MM-TFNet for edge-based family health monitoring, the computational complexity of MM-TFNET was quantitatively analyzed. The feature of this model is that it has approximately 2 million training parameters and occupies about 7.34 MB of storage space when using float32 precision. For real-time applications, when the batch size is 32, the average single-sample inference delay achieved by this model is 170.33 ms. Each epoch during the training process takes approximately 17.57 s. These indicators confirm that MM-TFNet has great potential in future edge devices or portable medical systems.

## 6. Discussion

Heart rate estimation based on BCG has garnered increasing attention due to its comfortable and non-invasive characteristics. In recent years, deep learning models have demonstrated superior performance compared to traditional signal processing techniques in this domain. This study introduces MM-TFNet, a hybrid neural network that processes synchronized BCG and PPG signals using parallel TCN and BiLSTM, followed by multi-head self-attention mechanisms to emphasize salient heartbeat features. A cross-modal attention mechanism operates at the feature level to dynamically fuse BCG’s mechanical vibration characteristics with PPG’s volumetric blood flow information, thereby enhancing heart rate estimation robustness and accuracy. Experimental results demonstrate that MM-TFNet achieves a low overall MAE of 0.88 BPM, indicating highly precise predictions.

However, several limitations warrant consideration. First, the current fusion architecture targets single-task heart rate estimation and does not accommodate multi-parameter monitoring requirements, such as respiratory rate tracking or arrhythmia detection. Second, the dataset comprises predominantly healthy individuals, which may limit the model’s generalizability to clinical populations, particularly patients with cardiovascular diseases. Third, as analyzed in Section 5, the model’s performance degrades for subjects with highly volatile heart rates (e.g., X1013). Although MM-TFNet effectively fuses complementary information, its ability to track abrupt physiological changes is constrained by the inherent nature of BCG and PPG signals. BCG signals during strong body movements can be severely corrupted, and PPG signals are susceptible to perfusion changes and motion artifacts. When both modalities are degraded simultaneously in such dynamic states, the fusion model lacks reliable information for precise heartbeat timing.

To address these limitations, integrating ECG information presents a viable avenue for future optimization of MM-TFNet. For instance, a multi-task learning framework could be established, incorporating ECG waveform reconstruction or R-wave detection as auxiliary tasks. This strategy would guide the model to focus on electro-mechanical oscillation features synchronized with the cardiac cycle, thereby enhancing its ability to track rapid physiological changes and reducing estimation errors in unstable cases such as X1013. Furthermore, this approach could facilitate exploration of other clinically relevant parameters, such as respiratory rate or heart rate variability. From an application perspective, our model features a relatively modest number of parameters, thus presenting significant potential for deployment on edge computing devices such as smart beds, smart pillows, or chairs. This is consistent with the growing trend in health monitoring, which involves shifting analysis from the cloud to the edge to ensure data privacy and real-time responsiveness.

## 7. Conclusions

This study presents MM-TFNet, a feature-level fusion hybrid neural network that combines BCG and PPG signals for heart rate estimation using non-invasive sensor data. The model integrates multi-scale convolution for local pattern extraction, TCN blocks for capturing long-range temporal dependencies, BiLSTM for sequential learning, and cross-modal attention for dynamic feature integration. A five-fold cross-validation strategy was employed during training, and the model achieved a competitive MAE of 0.88 BPM, outperforming conventional approaches and recent deep learning baselines. Future research will focus on optimizing computational efficiency and enabling deployment for edge-based, low-power inference. We aim to extend the model’s application to real-world scenarios, including elderly home monitoring, maternal-infant care, and sleep apnea screening. Through integration with wearable devices and portable medical systems, the proposed solution demonstrates potential to support real-time, non-invasive cardiovascular monitoring and remote early warning systems, providing a high-precision, low-latency platform for chronic disease management.

## Figures and Tables

**Table 1:** Comparison of different methods.

Category	Methods	Key Technology	Advantages	Limitations
Traditional Signal Processing	Brink [11]; Heise [12]; Xie [14]	Peak detection, Sliding window, Template matching	Simple, fast computation	Noise-sensitive, poor performance with motion
Machine Learning	Rosales [18]; Paalasmaa [19]	K-means clustering, Adaptive template learning	Adapt to individual waveforms	Needs feature engineering; struggles with non-stationary signals
Deep Learning (Single Modal)	Jiao [21]	Bidirectional LSTM regression	Learn from raw data; less reliant on annotations	Sensitive to strong artifacts
	Zhang [25]	CNN + Transformer	Capture local and global features; end-to-end	Higher model complexity
Multimodal Fusion	Warnecke [28]	CNN-based fusion of ECG, BCG, PPG, iPPG	Robust via multi-sensor redundancy	Focus on detection, not regression; complex setup

**Table 2:** Participant characteristics. SD: standard deviation. BMI: body mass index.

Variable	All (*n* = 40)		Males (*n* = 17)		Females (*n* = 23)	
	Mean (SD)	Range	Mean (SD)	Range	Mean (SD)	Range
Age (years)	34 (15)	18–65	32.4 (15.5)	19–65	35.3 (14.8)	18–65
Height (cm)	171 (11)	147.6–197.8	178.3 (11.1)	166.3–197.8	165.2 (10.3)	147.6–188.5
Weight (kg)	76 (18)	48.3–136	84.4 (13.5)	64–136	67.3 (15.6)	48.3–136
BMI (kg/m^2^)	26 (5.7)	18.4–51.7	26.9 (3.8)	21.4–48.2	25.3 (5.9)	18.4–51.7

**Table 3:** Ablation study: MAE Performance comparison across different model variants.

Variant Configuration	MAE (BPM)
Baseline	3.47
Baseline + TCN	3.27
Baselline + TCN + BiLSTM	1.37
MM-TFNet only with PPG	3.11
MM-TFNet without MF	2.12
MM-TFNet without PPG	1.51
MM-TFNet	0.88

**Table 4:** Comparison of heart rate prediction with different methods.

Method	Signals	Method	MAE (BPM)
Pröll et al., 2021 [23]	BCG	CNN + GRU	2.07
Jiao et al., 2021 [21]	BCG	BiLSTM	1.42
Zhang et al., 2023 [25]	BCG	CNN + Transformer	0.93
Our method	BCG + PPG	TCN + BiLSTM + Attention	0.88

## Data Availability

The data presented in this study are available from the corresponding author upon request.

## References

[1] Sadek I., Biswas J. (2018). Nonintrusive heart rate measurement using ballistocardiogram signals: A comparative study. Signal Image Video Process..

[2] Liu M., He X., Yang X., Wang Z. (2025). Interpretation of Report on Cardiovascular Health and Diseases in China 2023. Chin. Gen. Pract..

[3] Mensah G.A., Fuster V., Murray C.J.A., Roth G.A., Abate Y.H., Abbasian M., Abd-Allah F., Abdollahi A., Abdollahi M., Abdulah D.M. (2023). Global Burden of Cardiovascular Diseases and Risks, 1990–2022. J. Am. Coll. Cardiol..

[4] Sadek I., Biswas J., Abdulrazak B. (2019). Ballistocardiogram signal processing: A review. Health Inf. Sci. Syst..

[5] Kim K.B., Baek H.J. (2023). Photoplethysmography in Wearable Devices: A Comprehensive Review of Technological Advances, Current Challenges, and Future Directions. Electronics.

[6] Coste A., Millour G., Hausswirth C. (2025). A Comparative Study Between ECG- and PPG-Based Heart Rate Sensors for Heart Rate Variability Measurements: Influence of Body Position, Duration, Sex, and Age. Sensors.

[7] Sadek I., Abdulrazak B. (2021). A comparison of three heart rate detection algorithms over ballistocardiogram signals. Biomed. Signal Process. Control.

[8] Jung H., Kimball J.P., Receveur T., Agdeppa E.D., Inan O.T. (2021). Accurate ballistocardiogram based heart rate estimation using an array of load cells in a hospital bed. IEEE J. Biomed. Health Inf..

[9] Yousefian P., Shin S., Mousavi A.S., Tivay A., Kim C., Mukkamala R., Jang D.-G., Ko B.H., Lee J., Kwon U.-K. (2020). Pulse transit time-pulse wave analysis fusion based on wearable wrist ballistocardiogram for cuff-less blood pressure trend tracking. IEEE Access.

[10] García-Limón J.A., Alvarado-Serrano C., Casanella R. (2024). A Novel BCG Heart Rate Detection System Using a Piezoelectric Sensor Embedded in a Shoe Insole. IEEE Sens. J..

[11] Brink M., Müller C.H., Schierz C. (2006). Contact-free measurement of heart rate, respiration rate, and body movements during sleep. Behav. Res. Methods.

[12] Heise D., Skubic M. Monitoring pulse and respiration with a non-invasive hydraulic bed sensor. Proceedings of the 2010 Annual International Conference of the IEEE Engineering in Medicine and Biology Society.

[13] Heise D., Rosales L., Skubic M., Devaney M.J. Refinement and evaluation of a hydraulic bed sensor. Proceedings of the 2011 Annual International Conference of the IEEE Engineering in Medicine and Biology Society.

[14] Xie Q., Wang M., Zhao Y., He Z., Li Y., Wang G., Lian Y. (2019). A personalized beat-to-beat heart rate detection system from ballistocardiogram for smart home applications. IEEE Trans. Biomed. Circuits Syst..

[15] Brüser C., Winter S., Leonhardt S. (2013). Robust inter-beat interval estimation in cardiac vibration signals. Physio Gymea.

[16] Brüser C., Kortelainen J.M., Winter S., Tenhunen M., Pärkkä J., Leonhardt S. (2015). Improvement of Force-Sensor-Based Heart Rate Estimation Using Multichannel Data Fusion. IEEE J. Biomed. Health Inf..

[17] Feng J., Huang W., Jiang J., Wang Y., Zhang X., Li Q., Jiao X. (2023). Non-invasive monitoring of cardiac function through Ballistocardiogram: An algorithm integrating short-time Fourier transform and ensemble empirical mode decomposition. Front. Physio.

[18] Rosales L., Skubic M., Heise D., Devaney M.J., Schaumburg M. Heartbeat detection from a hydraulic bed sensor using a clustering approach. Proceedings of the 2012 Annual International Conference of the IEEE Engineering in Medicine and Biology Society.

[19] Paalasmaa J., Leppakorpi L., Partinen M. Quantifying respiratory variation with force sensor measurements. Proceedings of the 2011 Annual International Conference of the IEEE Engineering in Medicine and Biology Society.

[20] Cathelain G., Rivet B., Achard S., Bergounioux J., Jouen F. U-Net neural network for heartbeat detection in ballistocardiography. Proceedings of the 2020 42nd Annual International Conference of the IEEE Engineering in Medicine & Biology Society (EMBC).

[21] Jiao C., Chen C., Guo S., Hai D., Su B., Skubic M., Jiao L., Zare A., Ho K.C. (2021). Non-invasive heart rate estimation from ballistocardiograms using bidirectional LSTM regression. IEEE J. Biomed. Health Inf..

[22] Mai Y., Chen Z., Yu B., Li Y., Pang Z., Han Z. (2022). Non-Contact Heartbeat Detection Based on Ballistocardiogram Using UNet and Bidirectional Long Short-Term Memory. IEEE J. Biomed. Health Inf..

[23] Pröll S.M., Tappeiner E., Hofbauer S., Kolbitsch C., Schubert R., Fritscher K.D. (2021). Heart rate estimation from ballistocardiographic signals using deep learning. Physiol. Meas..

[24] Su B.Y., Enayati M., Ho K.C., Skubic M., Despins L., Keller J., Popescu M., Guidoboni G., Rantz M. (2019). Monitoring the relative blood pressure using a hydraulic bed sensor system. IEEE Trans. Biomed. Eng..

[25] Zhang M., Qiu L., Chen Y., Yang S., Zhang Z., Wang L. (2023). A Conv-Transformer network for heart rate estimation using ballistocardiographic signals. Biomed. Signal Process. Control.

[26] Giovangrandi L., Inan O.T., Wiard R.M., Etemadi M., Kovacs G. Ballistocardiography—A method worth revisiting. Proceedings of the 33rd Annual International Conference of the IEEE Engineering in Medicine & Biology Society (EMBC).

[27] John A., Redmond S.J., Cardiff B., John D. (2022). A multimodal data fusion technique for heartbeat detection in wearable IoT sensors. IEEE Internet Things J..

[28] Warnecke J.M., Boeker N., Spicher N., Wang J., Flormann M., Deserno T.M. Sensor fusion for robust heartbeat detection during driving. Proceedings of the 43rd Annual International Conference of the IEEE Engineering in Medicine & Biology Society (EMBC).

[29] Bai S., Kolter J.Z., Koltun V. (2018). An empirical evaluation of generic convolutional and recurrent networks for sequence modeling. arXiv.

[30] Carlson C., Turpin V., Suliman A., Ade C., Warren S., Thompson D.E. (2021). Bed-Based Ballistocardiography: Dataset and Ability to Track Cardiovascular Parameters. Sensors.

[31] Nanchen D. (2018). Resting heart rate: What is normal?. Heart.

[32] Qiu Y., Chen W., Yue L., Xu M., Zhu B. STCT: Spatial-temporal conv-transformer network for cardiac arrhythmias recognition. Proceedings of the Advanced Data Mining and Applications: 17th International Conference ADMA 2021.

